# NLRP3 Inflammasome Activation of Mast Cells by Estrogen *via* the Nuclear-Initiated Signaling Pathway Contributes to the Development of Endometriosis

**DOI:** 10.3389/fimmu.2021.749979

**Published:** 2021-09-22

**Authors:** Xinyue Guo, Xinxin Xu, Tiantian Li, Qin Yu, Jianzhang Wang, Yichen Chen, Shaojie Ding, Libo Zhu, Gen Zou, Xinmei Zhang

**Affiliations:** ^1^ Department of Gynecology, Women’s Hospital, School of Medicine, Zhejiang University, Hangzhou, China; ^2^ Department of Pharmacology, Ningbo Institution of Medical and Science, Ningbo, China

**Keywords:** endometriosis, estrogen, mast cells, NLRP3 inflammasome, interleukin-1β

## Abstract

Endometriosis is an estrogen-dependent gynecological disease. The pathogenesis of endometriosis remains controversial, although it is generally accepted that the inflammatory immune response plays a crucial role in this process. Mast cells (MCs) are multifunctional innate immune cells that accumulate in endometriotic lesions. However, the molecular mechanism by which estrogen modulates MCs in the development of endometriosis is not well understood. Here we report that estrogen can induce the expression of NOD-like receptor family pyrin domain containing 3 (NLRP3) through estrogen receptor (ER)-α *via* the estrogen responsive element (ERE) in MCs. Such transcriptional regulation is necessary for the activation of NLRP3 inflammasome and the production of mature interleukin (IL)-1β in MCs. Targeted inhibition of NLRP3 significantly restrained lesion progression and fibrogenesis in a mouse model of endometriosis. Collectively, these findings suggest that MCs contribute to the development of endometriosis through NLRP3 inflammasome activation mediated by nuclear-initiated estrogen signaling pathway.

## Introduction

Endometriosis is an estrogen-dependent inflammatory disease in which endometrium-like tissue is presented outside the uterine cavity, primarily on the pelvic peritoneum and ovaries (1). It affects approximately 10% of women in their reproductive years and is related to pelvic pain and infertility (2, 3). The current consensus is that the origin of ectopic endometrial tissue is retrograde menstruation, and that the peritoneal immune microenvironment plays a vital role in the further development of endometriosis (4–6).

One essential type of immune cell with altered function in the peritoneal environment of endometriosis is mast cells (MCs) (7, 8). MCs are widely acknowledged to be critical players during allergic reactions (9). However, increasing evidence suggests that MCs are also involved in inflammatory and autoimmune diseases through promoting vascular permeability, facilitation of immune responses, and modulation of fibrosis (10–12). Many recent studies support this theory, suggesting that endometriotic lesions contain an increased number of activated MCs, which may secrete several inflammatory mediators such as histamines and tumor necrosis factor (TNF)-α (13, 14). In our previous studies, the recruitment and degranulation of MCs in endometriosis were associated with a high concentration of local estrogen (15). Estrogen can not only enhance MCs degranulation upon allergen cross-linking of IgE, but also directly activate MCs (16–18). Further studies report that MCs activation by estrogen plays a crucial role in the development of endometriosis (19), yet its underlying molecular mechanisms require further investigation.

NOD-like receptor family pyrin domain containing 3 (NLRP3) is an intracellular receptor that senses foreign and endogenous danger signals (20). It interacts with apoptosis-associated speck-like protein (ASC) and caspase-1 to form the NLRP3 inflammasome complex. The complex functions as an inducer of the release of pro-inflammatory cytokines interleukin (IL)-1β and IL-18, while also promoting immune response and pyroptosis (21). Previous studies have determined the special role of NLRP3 inflammasome in the activation of MCs during the autoinflammatory response (22). Targeting the activation of NLRP3 and the production of IL-1β are considered efficacious anti-cytokine therapy options (23). However, the mechanisms by which NLRP3 inflammasome impacts the development of ectopic endometrium are still unclear.

In this study, we conducted a series of experiments to provide evidence that estrogen promoted NLRP3 expression in MCs *via* estrogen receptor (ER)-α, and that this process was mediated by an estrogen responsive element (ERE). Elevation of NLRP3 induced potassium ion efflux, which activated the NLRP3 inflammasome signaling pathway and up-regulates the production of mature IL-1β. We then identified that the functional IL-1β receptor IL1R1 was overexpressed in ectopic endometrial stroma. In addition, our *in vivo* data suggested that inhibiting NLRP3 inflammasome activation through the introduction of CY-09 could significantly reduce the development of endometriotic lesions in mice.

## Materials and Methods

### Ethical Approval

This study was approved by the Human Ethics Committee of the Women’s Hospital, School of Medicine, Zhejiang University (No. 20160114). Written informed consent was obtained from each patient. The privacy rights of patients were observed.

### Cell Culture

Mouse mast cell line P815 from ScienCell Research Laboratories (CA, USA) was cultured with RPMI-1640 (Gibco, USA) supplemented with 10% fetal bovine serum (FBS) and 1% Penicillin-Streptomycin (PS) (Thermo Fisher). The 293T cells were purchased from ATCC and were maintained in DMEM(Gibco) supplemented with 10% FBS and 1% PS. All these cells were maintained at 37°C, in 5% CO2, with the medium being changed every 2–3 days.

### Stable Transfection

The mouse ESR1-specific shRNA vectors (SH) and the negative control vectors (NC) were purchased from Obio Technology (Shanghai, China). The targeted sequences were: sh-ESR1 (5′- GCAAGCCCACTGTGTTCAA-3′), sh-NC (5′- CCTAAGGTTAAGTCGCCCTCG-3′). Lentivirus packaging and transduction were conducted according to the manufacturer’s instructions. P815 cells stably expressing sh-ESR1 or NC were enriched by puromycin selection for positive clones. The knockdown efficiency of shRNA was confirmed by quantitative PCR and western blot.

### RNA Sequencing Analysis

Total mRNAs of P815 cells (three duplicate samples in each NC and SH group) incubated with 100 pmol/mL β-estradiol and serum-free control for 24h were isolated. RNA sequencing using HiSeq 4000 was carried out by LC Biological Technology (Hangzhou, China) according to the manufacturer’s instructions. Primary sequencing data was produced by RNA sequencing and these were subjected to quality control. FPKM (fragments per kilobase of exon model per million mapped reads) was used to measure the abundance of gene expression. The differential expression range of |log2FC| ≥1 was used as the change threshold, and the *P* value < 0.05 was the standard for the differentially expressed genes between the comparison groups. Subsequently, those genes with large differences and high significance were selected, and underwent Kyoto Encyclopedia of Genes and Genomes (KEGG) pathway enrichment analysis. Finally, the consistency with the sequencing results was verified by real-time PCR.

### Quantitative Real-Time PCR

Total RNA was extracted from cells using a Total RNA Isolation Kit (Vazyme, Nanjing, China) and reversed using the PrimeScript Reverse Transcription (RT) reagent kit (Takara) according to the manufacturer’s recommendations. Specific primers used for amplification were synthesized by Generay (Shanghai, China). The sequencing of primers was as follows: Mouse GAPDH: CTTCAACAGCAACTCCCACTCTTCC (F), GGTTTCTTACTCCTTGGAGGCCATG (R); Mouse ESR1: AGGTGCCCTACTACCTGGAG (F), TCTTTCCGTATGCCGCCTTT (R); and Mouse NLRP3: ATTACCCGCCCGAGAAAGG (F), TCGCAGCAAAGATCCACACAG (R). Real-time PCR was performed with an Applied Biosystems 7900HT system (Applied Biosystems) using a SYBR Premix Ex TaqTM kit (Takara). For each sample, an average cycle threshold (Ct) value was calculated from triplicate wells, expressions of genes were normalized to the expression of glyceraldehyde-3-phosphate dehydrogenase (GADPH), and the fold change was determined using the 2^-△△^Ct method.

### Western Blotting

Samples were homogenized using RIPA buffer (Beyotime, Shanghai, China) with Protease and Phosphatase Inhibitor (Thermo Fisher) or Nuclear-Cytosol Extraction Kit (Fdbio science, Hangzhou, China). Proteins were separated in SDS-PAGE and then transferred to the PVDF membranes (Merck Millipore). After blocking with 5% bovine serum albumin, the membranes were incubated with antibodies raised against Estrogen Receptor alpha (1:1000, ab32063, Abcam, USA), PCNA (Proteintech, USA), NLRP3 (1:1000, ab263899, Abcam), cleaved caspase-1 (1:1000, 89332, Cell Signaling Technology), pro-caspase-1 (1:1000, ab179515, Abcam), IL-1β (1:1000, 12242, Cell Signaling Technology), ASC (1:1000, 67824, Cell Signaling Technology), and GAPDH (1:1000, Mab5465, MultiSciences, Hangzhou, China). After incubation with HRP-conjugated secondary antibody against rabbit IgG (1:10000, ab97051, Abcam) or against mouse IgG (1:10000, ab97023, Abcam), the membranes were detected using the ECL detection kit (Biological Industries) and imaged on the ImageQuant LAS 4000 Mini Biomolecular Imager (GE Healthcare Life Sciences, USA). The detected protein expression was quantified on band volume by Image J and normalized over GAPDH mean band intensity.

### Intracellular K^+^ Measurement

To assess the intracellular amount of potassium, P815 cells were first incubated for different time points with 100 pmol/mL β-estradiol and serum-free control. Cells from each treatment condition were seeded into 96-well plates (30000 cells/well, Bioland Scientific, USA) in quintuplicate and wells were loaded with 10 µM of the cell permeant potassium-binding benzofuran-isophthalate acetoxymethyl ester (PBFI-AM, fluorescent K binding probe, ab142804, Abcam) with 0.1% Pluronic F-127 (Yeasen Biotech, Shanghai, China). After incubation for 2 h at 37 °C, excess PBFI-AM was removed using PBS. P815 were plated and signal intensity was measured with Varioskan Flash (Thermo Scientific). Excitation ratios of fluorescence at 340 and 380 nm, measured at emission 500 nm were used to detemine the intracellular content of K^+^, according to the manufacturer-suggested protocol. Measurements were corrected for autofluorescence values and presented as percent of untreated control.

### ELISA Assays

IL-1β levels were measured in the cell culture supernatant and peritoneal fluid of BALB/c mice using a mouse IL-1β ELISA kit (Cusabio, Wuhan, China) according to the manufacturer’s guidelines.

### Immunohistochemical Staining

Sections of human paraffin-embedded ovarian endometriomas and endometrial tissues were deparaffinized and rehydrated, followed by heat-induced and enzymatic antigen retrieval. Sections were then blocked before overnight incubation of primary antibodies: mast cell tryptase (1:250, ab2378, Abcam) and NLRP3 (1:100, ab263899, Abcam). Fluorescent secondary antibodies (Alexa Fluor 488 goat anti-mouse, 1:500, ab150117; and Alexa Fluor 647 donkey anti-rabbit, 1:500, ab150075, Abcam) and 4′,6-diamidino-2-phénylindole (DAPI, ab228549, Abcam) were then used for detection. Images were acquired using an Olympus FV1200 confocal microscope (Olympus, Tokyo, Japan). In the figures, scale bars are indicated on respective magnified images for each experiment. For fluorescence immunocytochemical assays, P815 cells incubated with or without 100 pmol/mL β-estradiol (Sigma) were seeded on μ-Slide 8 well chamber slides (ibidi GmbH, Germany) in complete RPMI-1640 medium. Cells were fixed in 4% buffered formalin, blocked and permeabilized before incubation with primary NLRP3 antibody (1:100, ab263899, Abcam). Fluorescent secondary Alexa Fluor 647 donkey anti-rabbit antibody (1:500, ab150075, Abcam) was then used for detection. DAPI (Abcam) was used for cell nuclear staining.

For histochemical assays, the endometrial tissues were fixed in 4% formalin and then embedded in paraffin. The 3 µm tissue slides were incubated with diluted primary antibody against interleukin-1 receptor IL1R1 (1:500, ab106278, Abcam) and IL1R2 (1:50, DF6682, Affinity Biosciences) to detect the expression of IL-1β receptors in endometrial tissues. Reaction products were visualized using 3, 3-diaminobenzidine (DAB) solution, and the nuclei were counterstained with hematoxylin. The results were examined by two independent observers who were unaware of the background of samples and the immunohistochemical scores were graded as follows: the staining intensity (0 representing negative, 1 for weak staining, 2 for moderate staining, and 3 for strong staining) and the percentage of positive cells (0 represented staining of 0–5%, 1 for staining of 6%–25%, 2 for staining of 26%–50%, 3 for 51%–75%, and 4 for 76%–100%) were recorded, and the IRS was calculated by multiplying the two scores (24).

### Chromatin Immunoprecipitation Assay

ChIP assay was performed using a ChIP assay kit (Abcam) according to the manufacturer’s protocol. In detail, 3 × 10^6^ P815 cells with or without estrogen treatment were fixed with 1.1% formaldehyde at room temperature for 10 min. Cells were sedimented, washed, lysed, and sonicated to reduce DNA lengths to between 200 and 1000 bp. After sonication, the chromatin was immunoprecipitated with anti‐Estrogen Receptor alpha (2µg for 1 × 10^6^ cells, ab32063, Abcam) or anti‐histone H3 (2µg for 1 × 10^6^ cells, ab1791, Abcam) antibodies at 4°C overnight. After incubating with antibody binding beads, DNA was purified using Proteinase K. PCR was performed using specific primers: forward (5′- TGCCTCATTCAGGACACT -3′), and reverse (5′- TGCTTGGCTTTCTTTACAC -3′). To confirm the PCR results, DNA sequencing of PCR products was performed by TsingKe Biological Technology (Beijing, China).

### Luciferase Assay

The NLRP3 promoter-reporter construct and the mutant NLRP3 promoter fragment, cloned into the pGL4 basic plasmids, were purchased from Obio Technology. The luciferase-encoding NLRP3-promoter or mutant NLRP3-promoter, renilla reporter constructs (Promega), and mouse ESR1 plasmid (Obio Technology) were co-transfected into 293T cells using Hieff Liposomal Transfection (Yeasen Biotech). The pGL4-Basic firefly luciferase vector served as the negative control. After 24 h, luciferase activities were measured using the Duo-Lite Luciferase Assay System (Vazyme) according to the manufacturer’s instructions. Renilla activity was used to normalize luciferase reporter activity. Assays were performed on cells in five wells for each experiment to obtain an average count, and three independent biological replicates were assessed.

### Animals

Female BALB/C mice (age: 6–8 weeks), housed in an environment maintained at 21 °C ± 0.5 °C, under a 12h light/dark cycle, and with free access to food and water, were used in the experiments. This study was carried out in strict accordance with the National Institutes of Health Guide for the Care and Use of Laboratory Animals. The protocol was approved by the Committee on the Ethics of Animal Experiments of Zhejiang University (No. ZJU20200015).

Eight mice were randomly selected as donors. The remaining mice were randomly divided into three groups (eight in each group): the sham group and two recipient groups, the control group, and the drug administration group. Bilateral ovariectomy was performed 7 days before endometriosis induction, followed by subcutaneous injection of 0.5 μg estradiol benzoate dissolved in 60 μL corn oil every 5 days. Induction of endometriosis was performed as reported previously (25). The uterine horns of donors were dissected and cut into fragments <1mm^3^. Then we suspended the fragments in 0.9% sterile saline and injected them intraperitoneally into the 16 recipient mice. Mice in the sham group were injected with an equal volume of 0.9% sterile saline.

Mice in the sham and control groups were injected intraperitoneally with the vehicle control (10% DMSO, 40% PEG400, 5% Tween80, and 45% water), while the drug administration group was given the proven NLRP3 inhibitor CY-09 (MCE, USA) (*i.p.*) at a dose of 2.5 mg/kg once a day (26). The mice were anesthetized with inhalation of CO_2_ and euthanized by cervical dislocation after treatment for 14 or 21 days. Endometriotic lesions and peritoneal fluid were preserved for further experiments, and the size and weight of lesions were measured. We determined the volume of lesions according to the formula: = (length × width^2^)/2. Sections of mouse paraffin-embedded endometriosis lesions were first subjected to hematoxylin and eosin (H&E) staining to confirm the diagnosis of endometriosis. Masson’s trichrome staining was performed as previously described (27), and the areas of the collagen fiber (blue-colored) in proportion to the entire field of lesions were measured using ImageJ.

### Flow Cytometry

Mouse peritoneal cells were isolated as described previously with slight modifications (28). In short, we injected 3 ml of phosphate-buffered saline (PBS) and 2 mL of air into the peritoneal cavity. After carefully massaging the abdomen, the cell suspension was withdrawn from the peritoneal cavity using a Pasteur pipette and was collected in centrifuge tubes. The collected cell suspension was centrifuged at 300 × g for 5 min at 4°C. The pellet was resuspended in PBS and incubated with Leukocyte Activation Cocktail with GolgiPlug (2 μL/mL, 550583, BD Pharmingen, USA) for 5 h in a 37°C humidified CO_2_ incubator. Following activation, peritoneal cells were harvested, washed with Flow Cytometry Staining Buffer (eBioscience), and incubated with mouse Fc Block (1µg for 1 × 10^6^ cells, 553141, BD Pharmingen). Then the cells were stained with APC-conjugated anti-mouse CD117/c-KIT (1:100, 553356, BD Pharmingen) and FITC-conjugated anti-mouse FcϵRIα (1:400, 11-5898-82, eBioscience) antibodies. MCs were identified by double-positive expression of FcϵRI and CD117. Intracellular staining of IL-1β was performed using the Transcription Factor Buffer Set (BD Pharmingen) and eFluor 450-conjugated anti-mouse IL-1 beta antibody (1:40, 48-7114-82, eBioscience) in accordance with the manufacturer’s instructions. APC-conjugated Rat IgG2b kappa (1:100, 17-4031-81, eBioscience) and FITC- conjugated Armenian Hamster IgG (1:400, 11-4888-81, eBioscience) antibody served as the isotype control. Flow cytometric data were acquired on a BD FACSVerse flow cytometer with BD FACSuite software.

### Statistical Analysis

All data were included for statistical analyses using GraphPad Prism 7 (Graph Pad Software) and were presented as mean ± standard error of the mean (SEM). All experiments were performed at least three times. Data normality was evaluated using Shapiro-Wilk normality test. For comparisons between two groups, statistical analysis was performed by Unpaired Student’s t-test (two-tailed) unless otherwise indicated. For multiple groups, one-way analysis of variance (ANOVA) followed by Dunnett t-test was used for normally distributed variables and the Kruskal-Wallis test with Dunn’s multiple comparison test for variables with skewed distribution. *P values < 0.05 were considered statistically significant.

## Results

### The Expression of NLRP3 Was Increased by Estrogen Stimulation *via* ER-α in MCs

**Figure 1:**
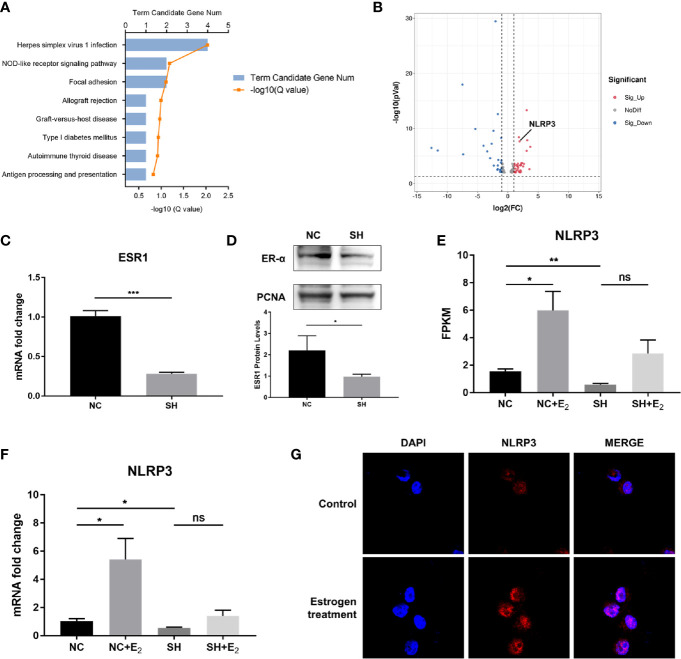
NLRP3 expression was increased by estrogen stimulation *via* ER-α in MCs. **(A)** Bar plot of differentially expressed genes (DEGs) from the KEGG pathway enrichment analysis; a comparison between β-estradiol treated P815 cells and the control. **(B)** A volcano plot was used for visualization of the differentially expressed genes. The gray point in the plot represents genes with no statistical differences (-1< log_2_(FC) < 1, *P* > 0.05), the red point represents upregulated genes (log_2_(FC) > 1, *P* < 0.05), and the blue point represents downregulated genes (log_2_(FC) < -1, *P* < 0.05) with statistical significance. **(C)** Quantitative PCR analysis of ESR1 in P815 cells after ESR1-specific shRNA transfection. **(D)** Western blot analysis of ER-α in P815 cell nuclei after stable transfection of ESR1-specific shRNA. **(E)** Relative quantification of NLRP3 mRNA level in P815 was measured by FPKM reads using RNA-seq. **(F)** Quantitative PCR analysis NLRP3 mRNA levels in P815 cells treated with β-estradiol after knockdown of ESR1 compared with the negative control. **(G)** Representative images of immunofluorescence staining for NLRP3 (red) in mast cell line P815 incubated with or without 100 pmol/mL β-estradiol for 24 h Data in **(C, D, F)** represent three independent experiments. Data are expressed as mean ± SEM. Statistics: Unpaired Student’s t-test. *P < 0.05, **P < 0.01, ***P < 0.001; ns, statistically not significant.

Essential roles of estrogen in orchestrating MCs activation have been established. In the quest to identify the underlying mechanisms that can coordinate MCs to participate in the inflammatory response in endometriosis, we first performed gene expression profiling to identify critical genes that responded to estrogen stimulation. We carried out KEGG enrichment pathway analysis of the differentially expressed genes (DEGs) between P815 cells treated with β-estradiol and the control. KEGG pathway analysis indicated that the DEGs were mainly involved in Herpes simplex virus 1 infection, NOD-like receptor signaling pathway, focal adhesion, allograft rejection, etc. (**Figure 1A**). Further study of DEGs revealed that NLRP3, an important molecule involved in regulating inflammation and the NOD-like receptor signaling pathway, was significantly increased in P815 after exposure to β-estradiol (**Figure 1B**). To gain insight into the molecular mechanism through which estrogen up-regulates the expression of NLRP3, we used ESR1-specific shRNA to silence ER-α in P815 cells. The efficiency of knockdown was verified by quantitative PCR, and western blot analysis confirmed that the protein level of ER-α in nuclei was significantly reduced (**Figures 1C, D**). RNA sequencing and quantitative PCR validated the elevated expression of NLRP3 in P815 cells transfected with negative control shRNA (NC) after incubation with β-estradiol, while the silencing of ER-α (SH) abrogated the effect (**Figures 1E, F**). Concurrent with the mRNA results, immunofluorescence staining of P815 cells revealed a pronounced increase of NLRP3 after β-estradiol treatment (**Figure 1G**). Collectively, these results suggested that the stimulation of estrogen may up-regulate the expression of NLRP3 in MCs.

### Upregulation of NLRP3 in MCs Was Induced by Estrogen Stimulation *via* the Interaction of ER-α With an ERE in the Promoter Region

**Figure 2:**
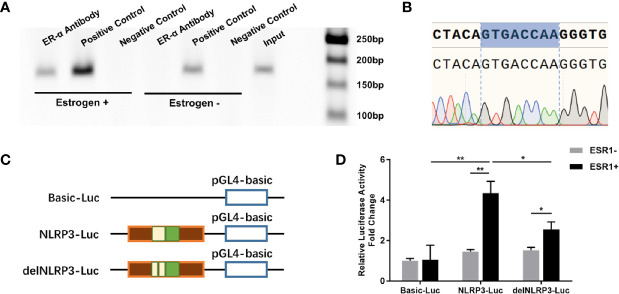
ERE sites in the NLRP3 promoter mediated estrogen-induced NLRP3 transcription. **(A)** ChIP analysis was performed using anti-ER-α or anti-Histone H3 antibody to ascertain the existence of the ERE in the promoter of the NLRP3 gene. The PCR results showed that a 159-bp fragment containing the presumed ERE could be precipitated after P815 cells were treated with β-estradiol for 24 h. **(B)** The pulled-down band was excised from the gel and sequenced. **(C)** Schematic diagram of luciferase reporter constructs. Basic-Luc: pGL4-basic plasmid; NLRP3-Luc: pGL4-basic plasmid with the NLRP3 promoter fragment including presumed ERE-like sequence; delNLRP3-Luc: pGL4-basic plasmid with the mutant NLRP3 promoter fragment, with the presumed ERE-like sequence deleted. **(D)** Luciferase activities of three report systems with or without ESR1 plasmid co-transfection in 293T cells were compared with each other. Renilla luciferase plasmid was used to normalize transfection efficiencies. The experiments were repeated three times and data are presented as means ± SEM. Statistics: Unpaired Student’s t-test. *P < 0.05, **P < 0.01.

In the search to define whether ER-α bound to the putative binding site, we analyzed the promoter sequence of the NLRP3 gene using online regulatory sequence analysis tools. Different primers were used to amplify the predicted ERE-like sequences. As shown in **Figure 2A**, one fragment containing the ERE-like sequence was captured using the ChIP assay after P815 cells were treated with estrogen. Specifically, after cell lysing and DNA shearing, extracts were subjected to ChIP-grade ER-α antibody and the anti-Histone H3 antibody (positive control). Following cross-linking reversal and protein digestion, PCR and agarose gel electrophoresis were carried out, and then gel extracted PCR products were subjected to gene sequencing. The DNA sequencing results confirmed the interaction of ER-α​ with the ERE (GTGACCAA) of NLRP3 positioned residues between −​422 through −​429 (**Figure 2B**). We further delved into the molecular mechanisms regulating ERE-dependent NLRP3 transcription using the dual-luciferase reporter assay. 293T cells were co-transfected with pGL4.10 reporter plasmid (Basic-Luc, NLRP3-Luc or delNLRP3-Luc), Renilla luciferase-expressing plasmid pRL-CMV, and plasmid pCMV-ESR1, as indicated (**Figure 2C**). Luciferase activities were measured after transfection to determine whether the putative ERE played a functional role in ER-α-related transcriptional activation. We found that the construct of the ERE (GTGACCAA) could interact with ER-α to activate the expression of downstream firefly luciferase, while the relative luciferase activity was significantly decreased when the construct of the partially deleted element was applied (**Figure 2D**). Our results suggested that a functional ERE motif existed in the NLRP3 gene promoter in MCs, and upregulation of NLRP3 was induced by estrogen stimulation *via* the interaction of ER-α with the ERE.

### NLRP3 Was Expressed in MCs in Endometriosis

**Figure 3:**
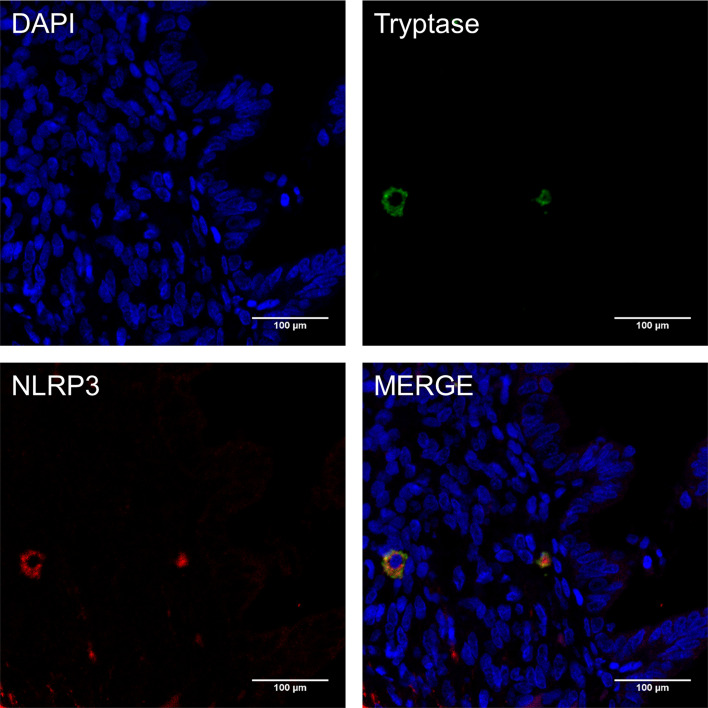
NLRP3 was expressed in mast cells in endometriosis. Immunofluorescence double staining for mast cell tryptase (green), NLRP3 (red) and cell nuclei (DAPI, blue) in paraffin sections of human ovarian endometriomas specimens (original magnification 800×; scale bars = 100 μm).

Previous studies demonstrate that MCs express functional components of the NLRP3 inflammasome (22). To directly address whether NLRP3 was expressed by MCs harbored in endometriotic lesions, double immunofluorescence was performed in paraffin sections of ovarian endometriomas. Merged images show the co-localization of NLRP3 (shown as red) and the mast cell marker, tryptase (shown as green; **Figure 3**), indicating that NLRP3 was expressed in MCs in endometriosis. Compellingly, the local high estrogen environment of endometriosis may lead to the changes of NLRP3 inflammasome in MCs, and its specific role in the development of the disease was worthy of our exploration.

### Estrogen Mediated the Activity of NLRP3 Inflammasome in MCs

**Figure 4:**
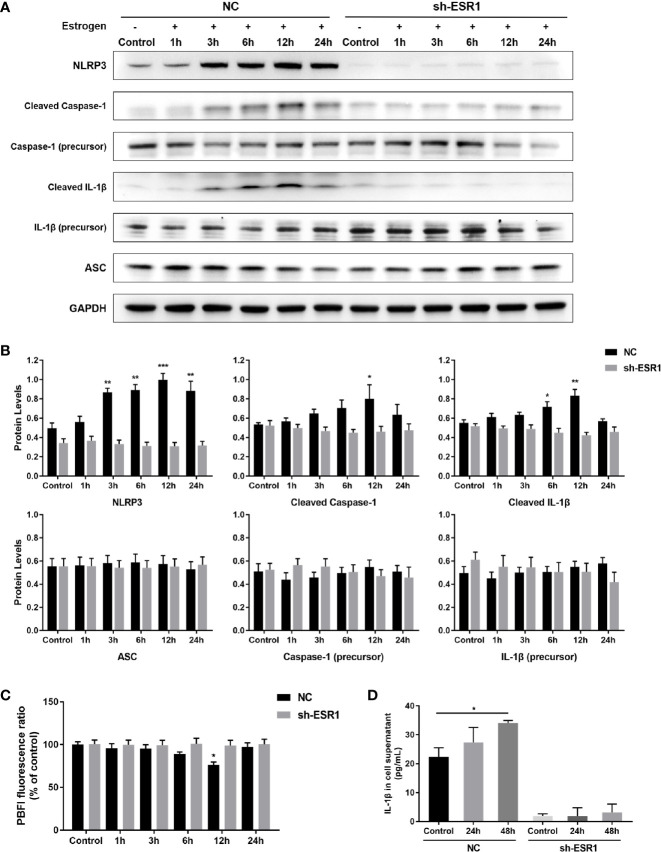
The NLRP3 inflammasome pathway was activated by estrogen *via* ER-α. **(A)** Western blot analysis of NLRP3, cleaved caspase-1, caspase-1 precursor, cleaved IL-1β, IL-1β precursor, and ASC in P815 cells treated with 100 pmol/mL β-estradiol for 1 h, 3 h, 6 h, 12 h, and 24 h. **(B)** Quantified results of western blot assays. **(C)** Intracellular concentration of K^+^ was determined by the ratio of the fluorescence intensities obtained by exciting PBFI at 340/380 nm wavelengths while monitoring emission at 500 nm, and presented as percent of untreated control. **(D)** The concentration of IL-1β in the P815 cell culture supernatant was tested using ELISA. Data in **(B–D)** represent three independent experiments. Data are expressed as mean ± SEM. Statistics: ANOVA followed by Dunnett t-test. *P < 0.05, **P < 0.01, ***P < 0.001.

Since the activation of caspase-1 is a key hallmark of the activity of the NLRP3 inflammasome pathway, we therefore assessed the protein expression level of cleaved caspase-1 and its target downstream molecule, IL-1β. Cleaved caspase-1 and IL-1β levels were significantly increased in NC groups treated with 100 pmol/mL β-estradiol for 12 h compared to the baseline measurements, while elevated NLRP3 levels were observed at 3 h, 6 h, 12 h, and 24 h after estrogen stimulation (**Figures 4A, B**). No difference between SH cells with or without estrogen treatment was observed at any time. We also evaluated the protein expression levels of ASC, pro-caspase-1, and pro-IL-1β and found that their expression did not change significantly after estrogen stimulation in either group. Since the activation of NLRP3 inflammasomes requires certain molecular and cellular signaling events (20), we further determined the intracellular K^+^ concentration of estrogen incubated P815 cells using fluorescent probe PBFI-AM at the aforementioned time points. Intracellular K^+^ was reduced in NC groups by 11% compared to control at 6 h after the start of incubation, with a maximum reduction of 24% observed at 12 h (**Figure 4C**), which indicated that K^+^ efflux occurred in MCs treated with estrogen. No significant difference in K^+^ concentration among the different treatments in SH groups was observed. ELISA assay of cell culture supernatant confirmed the increased mature IL-1β secretion level in the NC group when exposed to β-estradiol for 48 h (**Figure 4D**). The production of mature IL-1β was blunted in ER-α deficient MCs. These results clearly evidenced that estrogen could fuel the expression of NLRP3, and had a potent inflammasome activation capacity *via* K^+^ efflux in MCs.

### The Interleukin-1 Receptor Type 1 Was Predominant in Ectopic Endometrium

**Figure 5:**
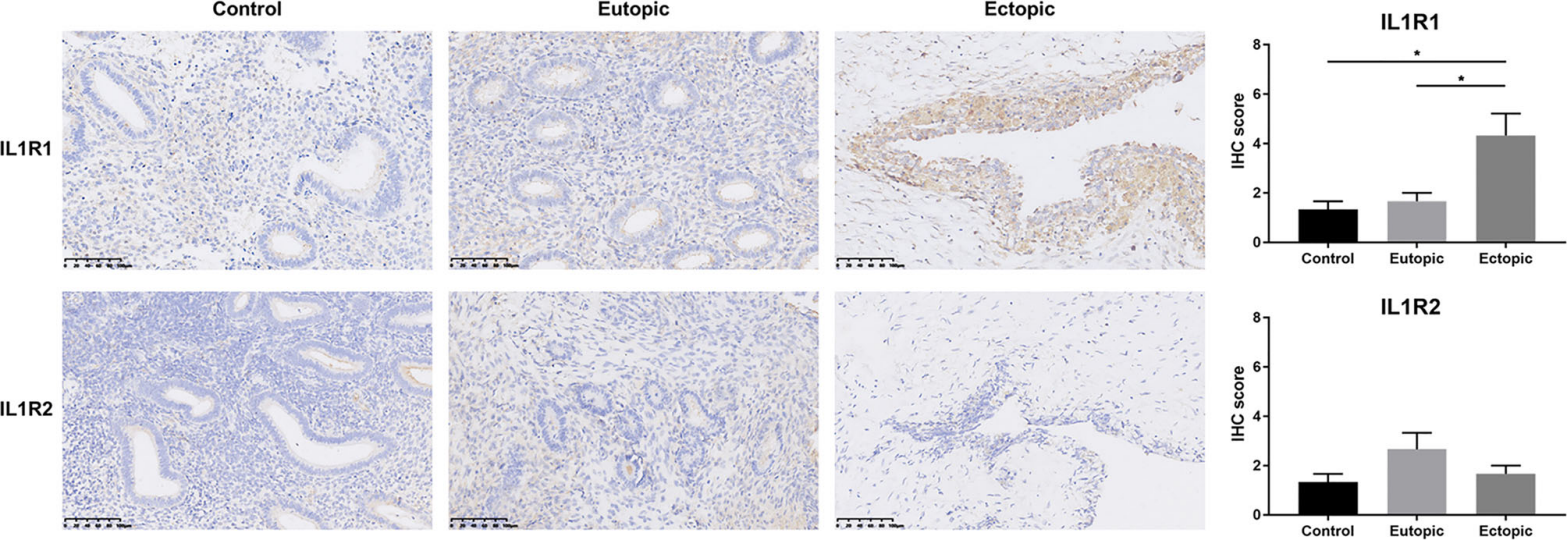
IL1R1 was over-expressed in ectopic endometrium. Representative photomicrographs of immunostaining with IL1R1 and IL1R2 in endometriotic lesions and eutopic endometrium from women with or without endometriosis. Scale bars = 100 μm. Immunostaining scores are represented as mean ± SEM. Statistical comparisons were carried out with Kruskal-Wallis test followed by Dunn’s multiple comparison test. *P < 0.05.

We have demonstrated that mature IL-1β was elevated in estrogen-stimulated MCs. Here, we tested the expression of its receptors in various types of endometrium. Immunohistochemical staining revealed a significantly elevated expression of IL1R1 in the ectopic endometrium, while the alternate receptor IL1R2 maintained low expression, consistent with previous reports (29) (**Figure 5**). Since IL1R2 is a recognized inhibitory receptor with no downstream signal transduction, IL-1β mainly combined with its functional receptor IL1R1 to promote the proliferation and invasive ability of endometrial stromal cells in the ectopic endometrium (30–32). These findings indicated a positive role for MC-derived IL-1β in steering the construction of the endometriosis lesions.

### Inhibition of NLRP3 Restricted Endometriotic Lesions Formation *In Vivo*

**Figure 6:**
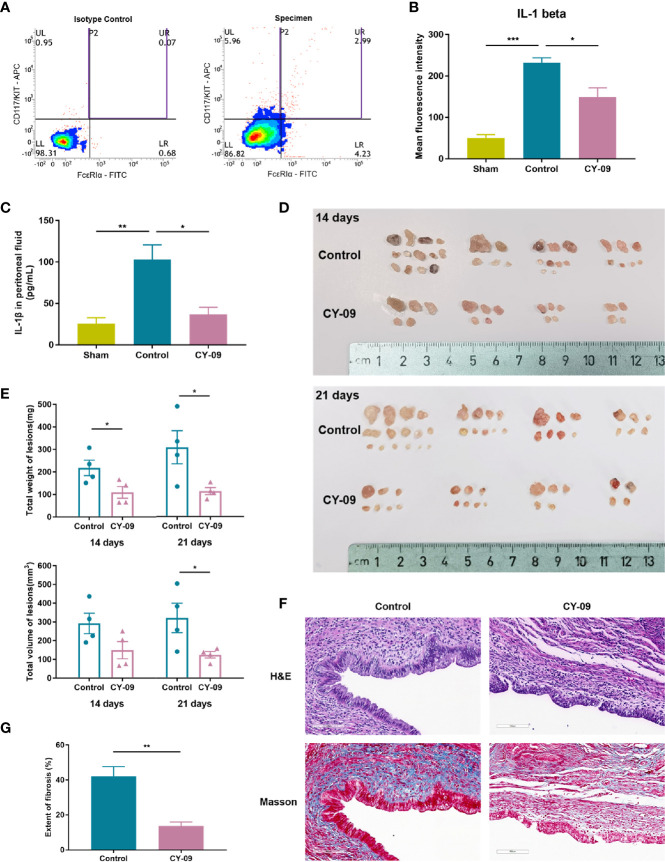
NLRP3 inhibitor CY-09 treatment suppressed the development of endometriotic lesions in mice. **(A)** Representative dot plot of cells in mouse peritoneal fluids by flow cytometric analysis using CD117/c-KIT and FcϵRIα staining. Mast cells were identified by double-positive expression of CD117 and FcϵRIα. **(B)** Peritoneal cells were stained for IL-1β, and the MFI of IL-1β in selected mast cells was analyzed by flow cytometric analysis. **(C)** The concentration of IL-1β in the peritoneal fluid of mice was tested using ELISA (n = 4). **(D)** Gross morphology of endometriotic lesions from mice of different groups. **(E)** The total weight and volume of endometriotic lesions in the control and CY-09 administration groups were assessed on day 14 and 21. **(F)** Representative hematoxylin and eosin (H&E) and Masson’s trichrome staining results in ectopic lesions from mice of different groups on day 21. Scale bar = 100 μm. **(G)** Quantified results of the extent of fibrosis in different groups. Results in **(B, C, E, G)** are expressed as mean ± SEM (n = 4 in each group). Statistics: Unpaired Student’s t-test. *P < 0.05, **P < 0.01, ***P < 0.001.

To investigate the role of MC-derived NLRP3 inflammasome in endometriosis, we intraperitoneally injected the NLRP3 inhibitor CY-09 into the established endometriosis mouse model. The level of IL-1β in peritoneal MCs was quantified using intracellular staining *via* flow cytometry. First, we identified MCs by double-positive staining of FcϵRIα and CD117 (**Figure 6A**). The average IL-1β mean fluorescence intensity (MFI) values in MCs from the control group were significantly higher than the sham and CY-09 administration group after 14 days of treatment (**Figure 6B**). Accordingly, IL-1β levels in the peritoneal fluid of mice with endometriosis were elevated compared with the sham group and were reduced when exposed to the NLRP3 inhibitor (**Figure 6C**). We further characterized the impact of NLRP3 inhibitor in the pathogenesis of endometriosis. A significant reduction of the total weight of endometriotic lesions was observed both at 14 and 21 days of CY-09 treatment (**Figures 6D, E**). The total volume of lesions in the CY-09 administration group was also significantly decreased over the control group at the time of sacrifice. Ectopic endometrial lesions obtained from the abdominal cavity of mice were verified by H&E staining, and fibrosis was assessed by staining sections with Masson’s trichrome (**Figure 6F**). Compared to the control group, CY-09 administration significantly reduced collagen deposition in endometriotic tissues (**Figure 6G**). Altogether, our data demonstrated a distinct alleviating role of NLRP3 inhibitor in the pathogenesis and fibrosis of endometriosis.

## Discussion

**Figure 7:**
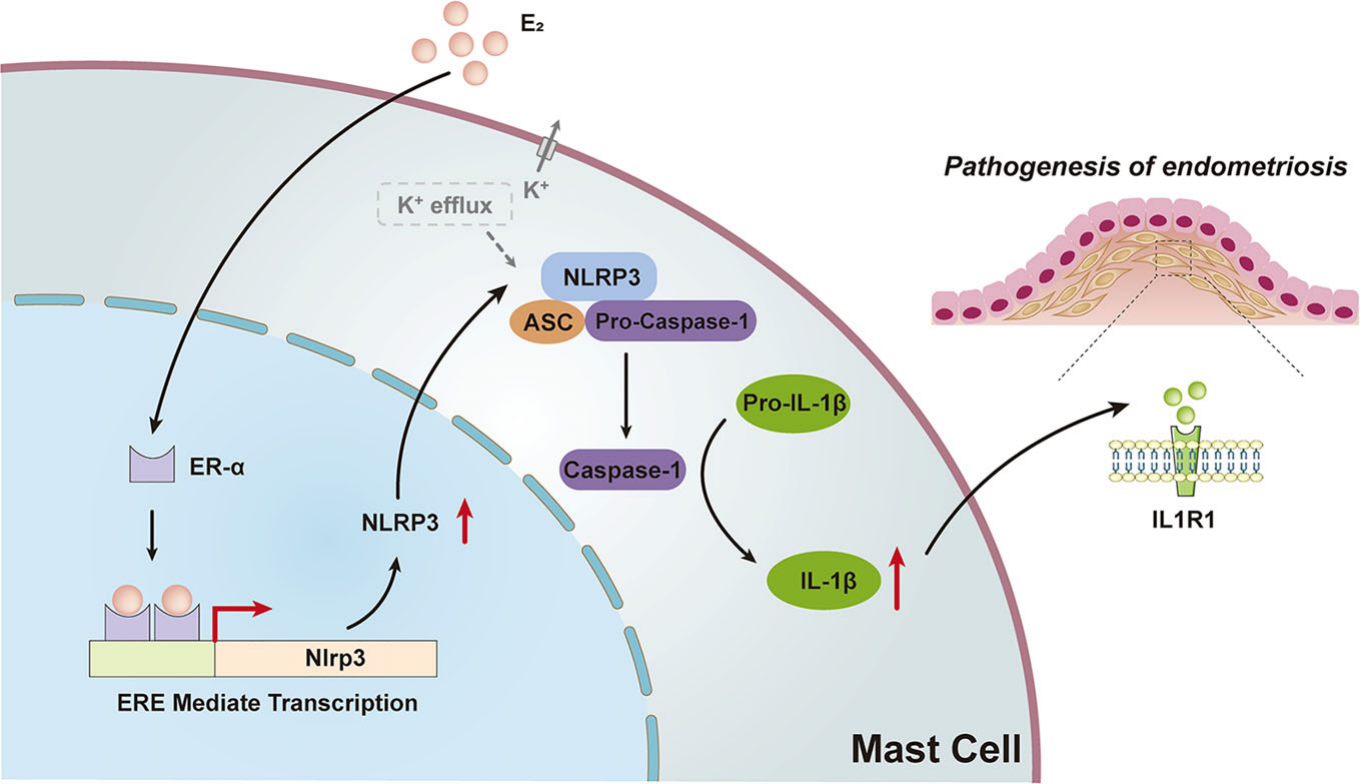
Schematic diagram of the present findings. High levels of estrogen in ovarian ectopic lesions can activate the NLRP3 inflammasome pathway by promoting ERE mediated transcription *via* ER-α in MCs. Increased expression of NLRP3 and K^+^ efflux activated the recruitment of inflammasomes, leading to the activation of caspase-1 and ultimately the production and secretion of functional IL-1β, which may trigger the pathogenesis of endometriosis.

The current study uncovered the molecular mechanisms that bridge the local high estrogen and MC pro-inflammatory response in the endometriotic lesions and identified NLRP3 inflammasome as an instrumental signaling pathway in perpetuating this local inflammatory response, as illustrated in **Figure 7**.

Estrogen regulated NLRP3 expression *via* ER-α in MCs. This finding has not been previously reported and is noteworthy as NLRP3 is a key factor necessary for immune defense and the pathogenesis of several inflammatory disorders (20). Previous studies demonstrate the classical priming steps of NLRP3 inflammasome initiated by microbial components or endogenous cytokines (33, 34). The priming signals mainly include caspase-8 and Fas-mediated death domain protein (FADD) interaction and transcription factor NF-κB activation (35, 36). Ultimately, NF-κB upregulates the expression of NLRP3 and licenses its activation (37). Notably, besides the transcriptional regulation of NLRP3, the activation signal (signal 2) provided by a variety of stimuli is considered indispensable for initiating inflammasome activation (38). It includes multiple upstream signals, most of which are not mutually exclusive, including ionic flux, lysosomal disruption, reactive oxygen species (ROS) and mitochondrial dysfunction, and metabolic changes, etc. Our data provided evidence that estrogen can directly interact with its nuclear receptor ER-α and promote the transcription of NLRP3 in the presence of ERE in MCs. In addition, the estrogen-induced K^+^ efflux was observed, which might be the signal critical for NLRP3 inflammasome activation, and silencing of ER-α attenuated this response. Our data surmise that estrogen acts on MCs most likely by promoting NLRP3 expression and K+ efflux responsible for the activation of NLRP3 inflammasome. Nevertheless, many reports emphasize the importance of NLRP3 inflammasome in the immune response of macrophages, with the role of NLRP3 in MCs being underestimated to date. Interestingly, NLRP3 inflammasome activation, which leads to inflammatory cytokine release and pyroptosis, has been shown to be involved in immune-related diseases, such as rheumatoid arthritis (RA), type 2 diabetes, atherosclerosis, and asthma (39–41). In these disorders, advances in MCs activation are positively associated with the aggravation of the status (42, 43). The persistent inflammatory response manifests that MCs can not only trigger an immediate and transient allergic reaction but continuously secrete inflammatory factors that accelerate the deterioration of the disease (44, 45). Several reports implicate the plasticity of MCs in specific tissues and adopted an NLRP3 inflammasome production role (46, 47). Our results indicate that the exposure of MCs to estrogen stably drives NLRP3 expression critical for caspase-1 activation and IL-1β secretion, which might contribute to the local proinflammatory environment in endometriosis. However, one potential concern is that we employed the murine mast cell line P815 for the *in vitro* experiments, as stated above. Further studies are warranted to investigate the transcriptional regulation mechanism of NLRP3 in a human mast cell line or primary mast cells, which might show some difference in the nuclear-initiated estrogen signaling pathway from that of murine cells. Also, our current investigation of intracellular ionic flux induced by estrogen in MCs is limited to observations on the phenomenon. It is necessary to further study the time effect of K^+^ efflux, the changes of K^+^ channels, and the impact of K^+^ efflux blockade on the activation of NLRP3 inflammasome. Another intriguing finding is that in the absence of estrogen, ER-α also seemed to have a promoting effect for NLRP3 transcription. This finding has to be interpreted with caution, and we believed it could be explored in future research to uncover the exact molecular mechanism by which ER-α can exert such an effect.

The current clinical trials for NLRP3 inflammasome-related diseases generally support the paradigm that targets IL-1β, mainly including the IL-1 receptor antagonist and IL-1β neutralizing antibody (48, 49). However, the production of IL-1β is regulated by many upstream signaling pathways. Pharmacological inhibition of IL-1β might dampen the inflammatory response and have more immunosuppressive effects, and also shadow the true pathogenic factor. In the meantime, other components of the NLRP3 inflammasome pathway, such as IL-18 and GSDMD, might also participate in the developing disease, and IL-1β inhibitor administration may fail to mitigate disease progression (50, 51). Our *in vivo* data supported that NLRP3 inhibitor CY-09 could suppress IL-1β production in MCs and protect against lesion development and fibrosis in endometriosis. Jiang et al. showed that CY-09 inhibited ATPase activity and oligomerization of NLRP3 by binding to its ATP-​binding Walker A motif (26). As such, CY-09 specifically inhibits the activation of NLRP3 inflammasome and has remarkable therapeutic effects in mouse models of some NLRP3-driven diseases like type 2 diabetes. Notably, recent concepts depicting the therapeutic role of IL-1β in endometriosis have emerged (52), raising the question of which one is the better treatment strategy, targeting IL-1β or NLRP3, or even a combined regimen. Further investigation is required to evaluate the pros and cons of these treatment strategies and define which can be most effective during endometriosis therapy. Also, future study on the targeting of NLRP3 inhibitors to MCs is warranted. The method of intraperitoneal injection of CY-09 adopted in this experiment may comprehensively impact various types of cells in the abdominal cavity of mice, including MCs, macrophages, and ectopic endometrial cells, etc. Although flow cytometry experiments have verified that the activity of NLRP3 inflammasomes in MCs was suppressed, other side effects have not been excluded. Therefore, the use of drug delivery carriers that precisely target certain cells will help determine the cellular function further and eliminate interference.

Taken together, our study provided mechanistic evidence of how estrogen can promote MC-derived NLRP3 inflammasome activation and IL-1β secretion, which lead to the development of endometriosis. We anticipate that improvements through targeted interventions to disrupt this process might hold promise for therapeutic strategies involving future drug intervention in the setting of clinical endometriosis.

## Data Availability Statement

The original contributions presented in the study are publicly available. This data can be found here: https://www.ncbi.nlm.nih.gov/geo/query/acc.cgi?acc=GSE181773.

## Ethics Statement

The studies involving human participants were reviewed and approved by the Human Ethics Committee of the Women’s Hospital, School of Medicine, Zhejiang University. The patients/participants provided their written informed consent to participate in this study. The animal study was reviewed and approved by the Committee on the Ethics of Animal Experiments of Zhejiang University. Written informed consent was obtained from the individual(s) for the publication of any potentially identifiable images or data included in this article.

## Author Contributions

XG and JW wrote the manuscript. XG and YC designed the experiments. XG, XX, TL, and QY performed the experiments and analyzed the data. XG, SD, LZ, and GZ compiled the figure**s**. JW and XZ reviewed the manuscript. All authors contributed to the article and approved the submitted version.

## Funding

This work was supported by the National Key R&D Program of China (Grant number: 2017YFC1001202) and the National Natural Science Foundation of China (Grant numbers: 81974225, 81671429, 81802591 and 81901459).

## Conflict of Interest

The authors declare that the research was conducted in the absence of any commercial or financial relationships that could be construed as a potential conflict of interest.

## Publisher’s Note

All claims expressed in this article are solely those of the authors and do not necessarily represent those of their affiliated organizations, or those of the publisher, the editors and the reviewers. Any product that may be evaluated in this article, or claim that may be made by its manufacturer, is not guaranteed or endorsed by the publisher.
